# Axillary Aneurism

**Published:** 1841-04

**Authors:** 


### AXILLARY ANEURISM.

The individual upon whom Professor Gross performed the operation of taking up the subclavian artery, on the 11th of February, died on the thirty-first day after the operation. The ligature came away on the fourteenth day, and the wound had entirely healed, without any bad symptoms.

A post-mortem examination revealed, as was expected, a perforation of the paries of the thorax, immediately contiguous to the aneurismal tumour, with a discharge of the contents of the sac into that cavity, and the necessary consequence of fatal pleuritis. The operation in itself was found to be perfectly successful; the artery being entirely obliterated at the point at which it was tied.

A full report of the case, together with a history of this operation, and some observations on the surgical anatomy of the parts, may be expected in an early number of the Journal.

